# Does Type of Tumor Histology Impact Survival among Patients with Stage IIIB/IV Non-Small Cell Lung Cancer Treated with First-Line Doublet Chemotherapy?

**DOI:** 10.1155/2010/524629

**Published:** 2010-07-20

**Authors:** Karen M. Clements, Gerson Peltz, Douglas E. Faries, Kathleen Lang, Joshua Nyambose, Craig C. Earle, Katherine P. Sugarman, Douglas C. A. Taylor, David Thompson, Martin D. Marciniak

**Affiliations:** ^1^i3 Innovus, Medford, MA 02155, USA; ^2^Global Health Outcomes, Lilly Corporate Center, Eli Lilly and Company, DC6831, Indianapolis, IN 46285, USA; ^3^Boston Health Economics, Inc., Waltham, MA 02451, USA; ^4^Institute for Clinical Evaluative Sciences, Toronto, ON, Canada M4N 3M5

## Abstract

Chemotherapy regimens may have differential efficacy by histology in nonsmall cell lung cancer (NSCLC). We examined the impact of histology on survival of patients (*N* = 2,644) with stage IIIB/IV NSCLC who received first-line cisplatin/carboplatin plus gemcitabine (C/C+G) and cisplatin/carboplatin plus a taxane (C/C+T) identified retrospectively in the SEER cancer registry (1997–2002). Patients with squamous and nonsquamous cell carcinoma survived 8.5 months and 8.1 months, respectively (*P* = .018). No statistically significant difference was observed in survival between C/C+G and C/C+T in both histologies. Adjusting for clinical and demographic characteristics, the effect of treatment regimen on survival did not differ by histology (*P* for interaction = .257). There was no statistically significant difference in hazard of death by histology in both groups. These results contrast the predictive role of histology and improved survival outcomes observed for cisplatin-pemetrexed regimens in advanced nonsquamous NSCLC.

## 1. Introduction

The American Cancer Society estimates that in 2008 there were 215,000 new cases of lung cancer and approximately 162,000 lung cancer deaths, making it the leading cause of cancer mortality in the United States (US) [1]. The most common form of the disease is non-small cell lung cancer (NSCLC), which accounts for approximately 75%–87% of all cases [2, 3]. The estimated 5-year survival rates are: 56% for stage I, 34% for stage II, 10% for stage III, and 2% for stage IV [1]. 

The three most common initial treatments for lung cancer are surgery, radiation therapy, and chemotherapy. Chemotherapy used alone is standard treatment for advanced or metastatic stage NSCLC (i.e., IIIB or IV). Platinum-based chemotherapy (e.g., cisplatin or carboplatin) has improved short-term survival, symptom control, and health-related quality of life for NSCLC patients in the last two decades [4–7]. Recent data from pemetrexed trials have shown that the effect of treatment on survival varied with histology in patients with advanced NSCLC [8–12]. These studies have investigated the treatment-by-histology interaction associated with pemetrexed-containing doublets or pemetrexed monotherapy. A phase III clinical trial in first-line NSCLC showed longer survival with cisplatin-pemetrexed in patients with nonsquamous cell carcinoma (HR = 0.81, *P* = .005) and shorter survival with cisplatin-pemetrexed in patients with squamous cell carcinoma (HR = 1.23, *P* = .05), compared to cisplatin-gemcitabine [13]. A differential effect for pemetrexed according to NSCLC histology has also been observed in phase 2 studies in advanced NSCLC [10, 11]. Several meta-analyses have been performed to compare cisplatin- and carboplatin-based chemotherapy in the treatment of advanced NSCLC [14–16]. Results have shown superiority in survival with cisplatin-based regimens that included third generation chemotherapy and superiority in terms of overall response rate although cisplatin-based regimens were associated with more severe toxicities. 

Doublet chemotherapy regimens generally consist of a platinum-based agent (cisplatin or carboplatin) given in combination with another 3rd-generation cytotoxic compound (e.g., a taxane, gemcitabine, or pemetrexed). In the event that primary chemotherapy treatment is unsuccessful, secondary treatments for advanced NSCLC patients may include pemetrexed, docetaxel, or erlotinib. However, none of these therapies are curative, and treatment outcome significantly depends on the size, type, and stage of the tumor and on the patient's state of health [17]. Median life expectancy for advanced NSCLC patients in whom one or more chemotherapy regimens has failed is approximately four months, during which time only palliative or supportive care is given (e.g., pain medication or oxygen) [4].

A study conducted by Ramsey et al. evaluated use, outcomes, and costs of alternative treatments among patients with stage IIIB or IV NSCLC [18]. Although the study provided a useful starting point for studying outcomes associated with NSCLC, it was based on older data (i.e., patients diagnosed between 1994 and 1999), and the role of histology was not examined in this study. Historically, few studies have examined the impact of histology on survival for patients with advanced stage NSCLC, and conclusions have been inconsistent regarding the prognostic or predictive role of histology in this population [19].

Recent data from three randomized, controlled phase III trials of pemetrexed regimens in advanced NSCLC have prompted a renewed interest in the impact of NSCLC histology on efficacy outcomes. A retrospective analysis of trials comparing second-line pemetrexed with docetaxel and a prospective analysis of a trial comparing first-line pemetrexed and cisplatin with gemcitabine and cisplatin identified statistically significant treatment-by-histology interactions, showing a survival advantage for nonsquamous cell carcinoma patients treated with pemetrexed regimens [8, 13]. In addition, a third randomized, placebo-controlled study investigating maintenance pemetrexed in advanced NSCLC confirmed the efficacy of pemetrexed in nonsquamous NSCLC [9]. Given these results and the lack of studies examining treatment-by-histology interaction in non-pemetrexed-containing chemotherapy regimens in patients with advanced NSCLC, the goal of this retrospective study was to evaluate whether or not histology predicted NSCLC survival outcomes in patients with advanced NSCLC with two commonly prescribed first-line doublet combinations, a platinum agent (cisplatin or carboplatin) plus gemcitabine (C/C+G) and a platinum agent plus a taxane (docetaxel/paclitaxel) (C/C+T), using data from the linked Surveillance, Epidemiology and End Results Program- (SEER-) Medicare database.

## 2. Methods

### 2.1. Data Source

The present study focused on patients who were identified through linked data from the SEER-Medicare database of the National Cancer Institute and administrative Medicare claims from the Centers for Medicare and Medicaid Services [20]. SEER cancer registry data from 1997 through 2002 were combined with Medicare claims from 1991 through 2003 for the analyses. Because Medicare participants are largely aged 65 and older, the sample was limited to this age group. Twelve registries participated in SEER-Medicare during 1997–1999 and 16 registries during 2000–2002, representing 14% and 25% of the US population, respectively. The combination of SEER and Medicare data provides information about demographics, comorbidities, initial cancer diagnosis, and treatment, as well as longer-term medical care. The combined dataset facilitates the linking of stage of diagnosis information with types and lengths of treatments from time of diagnosis through death [21].

### 2.2. Sample Selection and Followup

Patients who were newly diagnosed with stage IIIB/IV NSCLC between 1997 and 2002 were included in the study. The date of first NSCLC diagnosis in the SEER-Patient Entitlement and Diagnosis Summary File (PEDSF) was used as the index date for each patient. Patients were excluded if they were less than 65 years old; were enrolled in a health maintenance organization (HMO) at any time from 12 months prior to the index date through followup; were not eligible for Medicare Part A or B benefits from 12 months prior to the index date through followup; had any other cancer diagnosis prior to the index date; had Medicare eligibility based on end stage renal disease; had incomplete data (e.g., unknown month of cancer diagnosis); had a difference in date of death of 3 months or more as recorded by SEER and Medicare; or a NSCLC diagnosis date at death or autopsy. Study patients were followed to evaluate outcomes from their index date until either death or the end of the Medicare claims data (i.e., December 31, 2003), whichever came first.

Patients who received C/C+G and C/C+T first-line doublet chemotherapy regimens were included in the sample. This patient group was chosen because these doublet regimens were the most common first-line doublet chemotherapy regimens in the dataset, received by 84% of patients on any identifiable doublet chemotherapy. The doublet regimens were identified from Healthcare Common Procedure Coding System codes on Medicare claims. The earliest chemotherapy regimen that occurred during an initial treatment period (within 30 days prior to and 90 days post-index date), was considered to be the first-line treatment. The 30 days prior to the index date in the initial treatment period was intended to account for any lag time in the recording of the NSCLC diagnosis in the SEER registry. The date of treatment was defined as the date of the first chemotherapy claim, and multiple courses of the same chemotherapy agent were considered to be part of the first-line treatment. If a second chemotherapy agent was received within eight days of the first agent, then it was considered to be part of the first-line treatment (i.e., doublet regimen). 

### 2.3. Study Measures

#### 2.3.1. Histology

Patients were stratified by NSCLC histology and were further categorized as having squamous or nonsquamous cell carcinoma. This classification was chosen based on the results of previous studies that demonstrated survival advantages associated with nonsquamous cell histology compared to squamous cell histology. The squamous cell carcinoma category included large-cell squamous cancers and keratinizing and nonkeratinizing squamous cancers (ICD-0-2 histology codes: 8070/3, 8071/3, 8072/3). The nonsquamous cell carcinoma category included large cell nonsquamous cancers, adenocarcinomas, and NSCLCs with other/mixed histologies (ICD-0-2 histology codes: 8012/3, 8140/3, 8480/3, 8481/3, 8490/3, 8560/3, 8570/3). 

#### 2.3.2. Demographic and Clinical Characteristics

The SEER-Medicare database contained information on gender, race, geographic region (Midwest, Northeast, South, and West), location of residence (Metropolitan, Urban, and Rural), mean household income by zip code, and cancer stage at diagnosis. The Charlson Comorbidity Index score [22] was calculated from Medicare claims during the 12-month pre-index period using the Deyo [23] adaptation of the scale for use with ICD-9-CM diagnosis codes excluding conditions likely to be related to NSCLC (i.e., chronic lung disease and malignancy).

### 2.4. Data Analysis/Statistical Methods

#### 2.4.1. Descriptive Statistics

Patients with stage IIIB/IV NSCLC were stratified by histology and described according to baseline clinical and demographic characteristics. 

#### 2.4.2. Survival

Survival was evaluated in terms of months from the index date until death with patients whose followup ended prior to death considered censored. Analyses comparing survival by histology among patients treated with C/C+T and C/C+G were conducted both unadjusted and adjusted for demographic and baseline clinical characteristics. Overall survival was compared by histology group. Within each histology group, the survival distribution was estimated using the Kaplan-Meier estimator, and the association between histology and survival was assessed with the log-rank test. A second analysis that adjusted for measured confounders using a Cox model was conducted. In the Cox model analysis, hazard ratios for each covariate represent the adjusted risk of death among patients with the covariate compared to patients in the respective referent categories (females, stage IIIB, non-Hispanic Caucasian, Charlson Comorbidity Index ≤1, Eastern geographic region, suburban location of residence, squamous cell carcinoma, and C/C+G). The model included a term for histology to assess whether histology was associated with survival and a treatment-by-histology interaction term to formally test whether the association between treatment and survival differed by histology. While used to minimize biases due to confounding factors, this modeling approach does not address the potential for confounding due to factors that were not available to us in the SEER-Medicaid database and relies on appropriate modeling and other statistical assumptions [24].

## 3. Results

**Figure 1:**
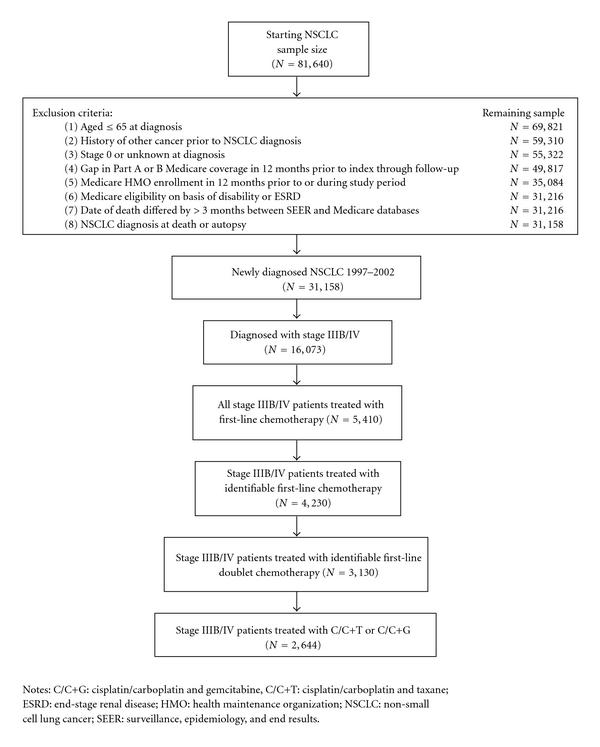
Flow of patients with Stage IIIB/IV who received cisplatin/carboplatin plus taxane (C/C+T) or cisplatin/carboplatin plus gemcitabine (C/C+G) first-line doublet chemotherapy from identification to sample selection.

From an initial sample of 81,640 patients newly diagnosed with NSCLC between 1997 and 2002, 31,158 met all initial study inclusion criteria (Figure 1). Approximately 34% (*n* = 10,475) were diagnosed at Stage IV, and 18% (*n* = 5,598) were diagnosed at Stage IIIB. Of these, 5,410 patients received first-line chemotherapy, 4,230 of whom had records allowing for identification of a specific chemotherapy regimen, and 3,130 of whom received a doublet regimen. Of these, 2,644 patients received C/C+T or C/C+G. This last group comprised the final analytic sample (Figure 1). Descriptive statistics for the baseline patient characteristics stratified by histology are shown in Table 1. The mean age of the stage IIIB/IV patients was 73 years old, collapsing across histology. A majority of patients were male (68.7% in the squamous cell carcinoma group and 55.5% in the nonsquamous cell carcinoma group) and most were non-Hispanic Caucasians, (90.4% in the squamous cell carcinoma group and 91.0% in the nonsquamous cell carcinoma group). A greater number of patients lived in the western region of the US (31.8% in the squamous cell carcinoma group and 40.2% in the nonsquamous cell carcinoma group) compared to the three other geographic regions, and most patients lived in a metropolitan area (85.2% collapsing across histology). The mean Charlson Comorbidity Index score excluding chronic obstructive pulmonary disease and cancer was 0.19 ± 0.6 in the squamous cell carcinoma group and 0.18 ± 0.5 in the nonsquamous cell carcinoma group.

**Figure 2:**
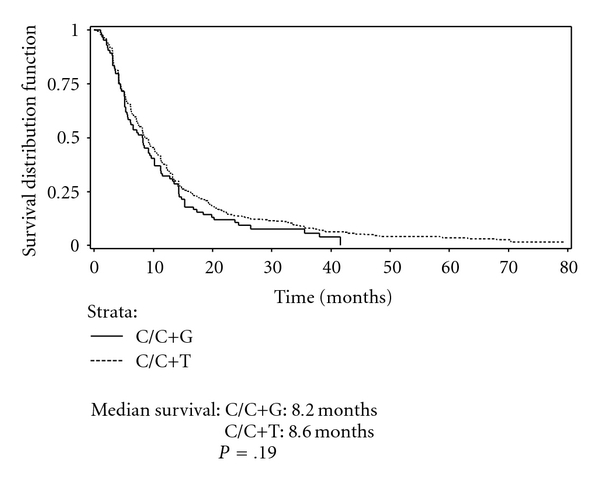
Kaplan-Meier survival curve of patients on doublet chemotherapy, by treatment, stage IIIB/IV, squamous.

**Figure 3:**
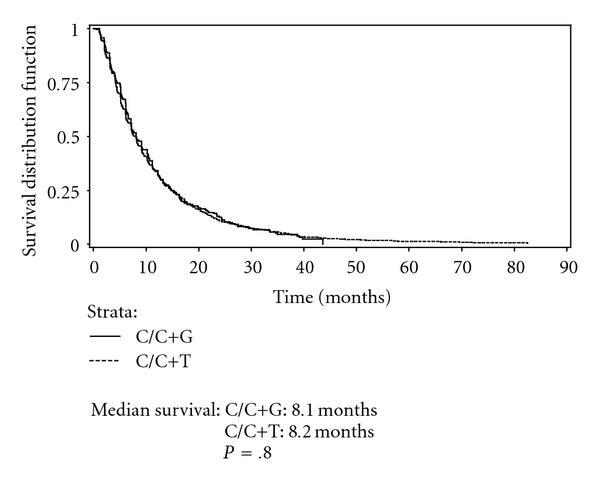
Kaplan-Meier survival curve of patients on doublet chemotherapy, by treatment stage IIIB/IV, nonsquamous.

**Figure 4:**
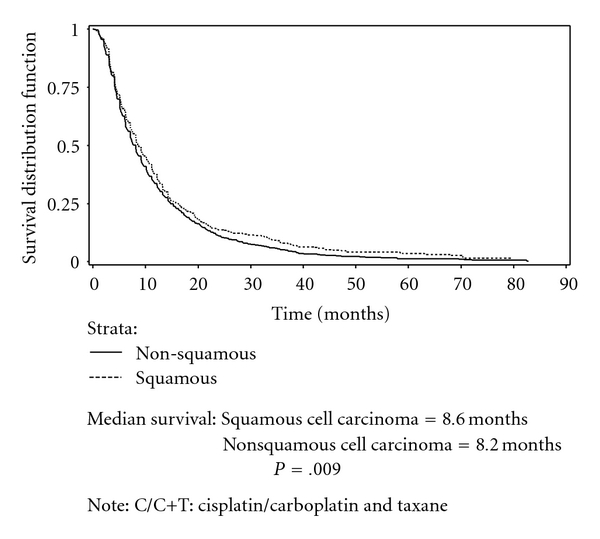
Kaplan-Meier survival curve of patients on doublet chemotherapy, by histology, stage IIIB/IV, C/C+T.

**Figure 5:**
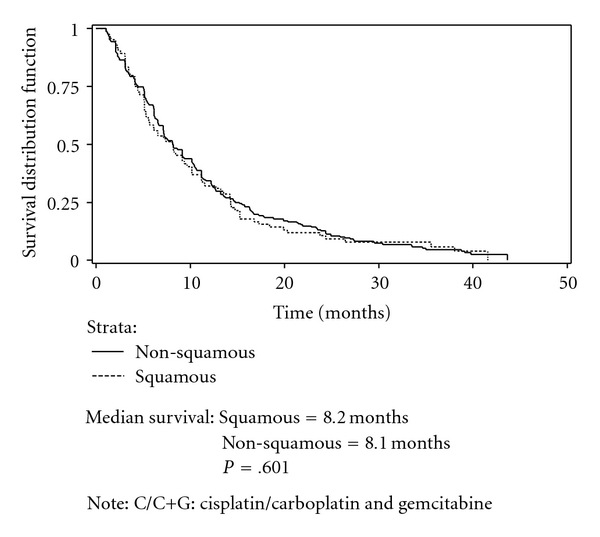
Kaplan-Meier survival curve of patients on doublet chemotherapy, by histology, stage IIIB/IV, C/C+G.

Figures 2 and 3 present Kaplan-Meier curves comparing survival among patients receiving C/C+T and C/C+G by histology. No significant difference in survival between therapies was found in either the nonsquamous cell carcinoma group or squamous group. Figures 4 and 5 present the Kaplan-Meier curves comparing survival between histology groups among patients receiving C/C+T and C/C+G, respectively. Overall, median survival of patients with squamous cell carcinoma was higher than for patients with nonsquamous cell carcinoma (8.5 months and 8.1 months, resp.; *P* = .018). Among patients receiving C/C+T, the median survival was 8.6 months and 8.2 months for patients with squamous cell carcinoma and nonsquamous cell carcinoma, respectively (*P* = .009). Among patients receiving C/C+G, the median survival was 8.2 months and 8.1 month for squamous cell carcinoma and nonsquamous cell carcinoma, respectively (*P* = .601).

Table 2 shows the Cox proportional hazard ratios for the characteristics associated with death for patients who received C/C+G and C/C+T as first-line treatment. Increased risk of death was observed for older age (HR = 1.009, 95% CI = 1.001–1.018 for each additional year), male sex (HR = 1.31, 95% CI 1.21–1.42), and stage IV (HR = 1.38, 95% CI 1.27–1.49). Increased risk of death was observed for nonsquamous cell histology (HR = 1.14, 95% CI 1.03–1.25). Survival did not differ by treatment regimen (*P* = .29), nor did treatment regimen affect the association between histology and survival (*P* = .26). Thus, while C/C+T in squamous patients had longer median survival than C/C+G by histology type combinations, there was no sufficient evidence in this sample to declare a statistically significant interaction.

## 4. Discussion

The present study examined the relationship between histology and survival in patients 65 years of age and older with stage IIIB/IV NSCLC receiving doublet chemotherapy regimens. Combining SEER and Medicare data provides information on both initial cancer diagnosis and later cancer treatment. SEER-Medicare data have been used in several published studies of a variety of cancers, including cancers of the breast, prostate, lung, and colon [20]. The combined source is appealing for cancer studies due to its size, comprehensiveness, and accessibility.

The median survival associated with the use of C/C+G and C/C+T in our study was lower than what has been observed in clinical trials [12, 25]. We speculate that such differences may be explained threefold. First, the mean age of participants in our study was 73 years compared to a range of 62–64 years observed in most clinical trials not restricted to an older population; second, due to data limitations, we were unable to stratify or exclude patients based on key baseline characteristics such as performance status (PS), organ function, or comorbidities; third, patients included in this investigation may not have had the same strict followup as dictated in controlled clinical trials. 

To our knowledge, this is the first study that examined treatment-by-histology interaction in nonpemetrexed-containing chemotherapy regimens for patients with advanced stage NSCLC. A systematic review of the published literature has shown that very few studies in advanced NSCLC have examined a formal treatment-by-histology interaction test [19]. Our study, which included a formal test of histology interaction, suggested that histology did not predict any significant differences in survival with C/C+G or C/C+T regimens, such that no survival advantages were identified for these regimens in either histology subgroup. We did observe a slightly increased risk of death associated with nonsquamous cell histology both overall and with patients treated with C/C+T. However, the generalizability of these results is limited to patients aged 65 years and older with advanced NSCLC receiving C/C+G or C/C+T.

This study is not without limitations. First, we partially relied on administrative Medicare claims data for patients aged 65 years and older. Medicare claims are primarily used for administrative purposes in obtaining reimbursement for services and not for research purposes. SEER data are limited in the amount of clinical and baseline information; therefore, we were unable to examine prognostic variables such as weight loss, smoking status, and PS as well as to evaluate the impact of the treatment on quality of life. Furthermore, patients enrolled in other forms of coverage than Medicare were excluded from the analyses. To the extent that patients aged less than 65 years may receive different treatments than older patients, our results may not be applicable to younger NSCLC patients. SEER-Medicare data are subject to some additional limitations, including that patients from SEER registries may not be representative of all US patients with NSCLC and certain groups are under- or over-represented (e.g., African-Americans and “other” races, resp., [8, 9]). Also, the algorithm developed to identify first-line therapy considered any chemotherapy administration that occurred within 8 days of the first drug to be a component of first-line treatment. Therefore, such algorithm increases the chances of missing those regimens given on a 21-day schedule, as well as those patients who experienced dose delays. Small sample sizes in certain histological subgroups precluded further separation of histology subgroups. As in any retrospective analysis, it is important to keep in mind that we are reporting claims data which is subject to errors in coding of diagnoses and procedures. Lastly, caution must be taken when interpreting comparative results from such naturalistic data as one cannot be certain that all confounding factors such as smoking history and PS have been accounted for. Additional data to test the replication of these findings would be of great value. 

Our findings provide increased understanding of survival among patients with stage IIIB/IV NSCLC, highlighting the role of histology in relationship to survival and chemotherapy. Median survival was longer for patients with squamous cell histology relative to the nonsquamous group. Whereas the C/C+T chemotherapy did have numerically longer median survival than the C/C+G in patients with squamous histology, the unadjusted Kaplan-Meier analysis directly comparing therapies within the squamous patients was nonsignificant. In addition, in the Cox model, the lack of a significant treatment effect and the lack of a significant treatment by histology interact suggest that there is not enough evidence in this sample to claim a differential treatment effect overall or between the histology groups. The lack of strong evidence showing differential treatment effects for C/C+T versus C/C+G in either histology group contrasts the results of clinical trials in patients treated with pemetrexed-containing regimens. These findings suggest that histology should be considered in the selection of treatment for patients with advanced NSCLC, particularly in regimens containing pemetrexed. Further studies are needed to confirm these findings. 

## Figures and Tables

**Table 1:** Baseline characteristics for 2,644 patients with stage IIIB/IV NSCLC treated with C/C+G or C/C+T, first-line doublet chemotherapy, by histology, SEER-Medicare Data 1997–2003.

	Histology	
	Squamous	Nonquamous
Characteristic		

*Patients*, *n* (%)	757 (28.6)	1,887 (71.4)

*Doublet*, *n* (%)		
C/C+G	84 (11.1)	289 (15.4)
C/C+T	673 (88.9)	1,598 (84.7)

*Age as of initial NSCLC diagnosis*:		
Mean (SD)	73 (4.6)	73 (4.7)
Median	72	73
Interquartile range	69–76	69–76

*Age-group as of initial NSCLC diagnosis*, *n* (%)		
65–74 years	507 (67.0)	1,196 (63.4)
75+ years	250 (33.30)	691 (36.6)

*Male*, *n* (%)	520 (68.7)	1,047 (55.5)

*Race*, *n* (%)		
Non-Hispanic Caucasian	684 (90.4)	1,718 (91.0)
Other	73 (9.6)	169 (9.0)

*Geographic region*, *n* (%)		
Midwest	169 (22.3)	345 (18.3)
Northeast	157 (20.8)	393 (20.8)
South	190 (25.1)	391 (20.7)
West	241 (31.8)	758 (40.2)

*Location of residence*, *n* (%)		
Metropolitan	628 (83.0)	1.626 (86.2)
Other	129 (17.0)	261 (13.8)

*Charlson comorbidity index, mean (sd)*	0.19 (0.57)	0.18 (0.52)

Notes: C/C+G: cisplatin/carboplatin and gemcitabine; C/C+T: cisplatin/carboplatin and taxane; NSCLC: non-small cell lung cancer; SD: standard deviation; SEER: surveillance, epidemiology, and end results.

**Table 2:** Characteristics associated with death following diagnosis of NSCLC, stage IIIB/IV for patients receiving C/C+G or C/C+T first-line chemotherapy.

Characteristics *N* = 2,644	95% CI			
	Hazard ratio	Lower limit	Upper limit	*P*
*Age at diagnosis*	1.009	1.001	1.018	.0339

*Sex*				
Male	1.310	1.207	1.422	<.0001
Female	1.00 (Referent)			

*Stage at diagnosis*				
IV	1.376	1.266	1.495	<.0001
IIIB	1.00 (Referent)			

*Race*				
Non-Hispanic Caucasian	1.069	0.932	1.226	0.3374
Other	1.00 (Referent)			

*Comorbidity*				
Charlson > 1	1.204	0.991	1.464	0.0617
Charlson ≤ 1	1.00 (Referent)			

*SEER registry region*				
Northeast	0.999	0.897	1.113	0.9878
Midwest	1.044	0.935	1.166	0.4467
South	1.036	0.930	1.153	0.5253
West	1.00 (Referent)			

*Location of residence*				
Metro	1.001	0.893	1.121	0.9916
Other	1.00 (Referent)			

*Histology*				
Nonsquamous cell	1.138	1.034	1.251	0.0079
Squamous cell	1.00 (Referent)			

*Primary treatment*				
C/C+G	1.137	0.900	1.436	0.2825
C/C+T	1.00 (Referent)			

*Histology∗treatment interaction*				
Nonsquamous∗C/C+G	0.857	0.656	1.119	0.2561

Notes: C/C+G: cisplatin/carboplatin and gemcitabine, C/C+T: cisplatin/carboplatin and taxane.
